# The Highs and Lows of FBXW7: New Insights into Substrate Affinity in Disease and Development

**DOI:** 10.3390/cells12172141

**Published:** 2023-08-24

**Authors:** Claire C. de la Cova

**Affiliations:** Department of Biological Sciences, University of Wisconsin-Milwaukee, Milwaukee, WI 53211, USA; delacova@uwm.edu

**Keywords:** FBXW7/FBW7/Cdc4/SEL-10, E3 ubiquitin ligase, phospho-degron, ubiquitin–proteasome system

## Abstract

FBXW7 is a critical regulator of cell cycle, cell signaling, and development. A highly conserved F-box protein and component of the SKP1–Cullin–F-box (SCF) complex, FBXW7 functions as a recognition subunit within a Cullin–RING E3 ubiquitin ligase responsible for ubiquitinating substrate proteins and targeting them for proteasome-mediated degradation. In human cells, FBXW7 promotes degradation of a large number of substrate proteins, including many that impact disease, such as NOTCH1, Cyclin E, MYC, and BRAF. A central focus for investigation has been to understand the molecular mechanisms that allow the exquisite substrate specificity exhibited by FBXW7. Recent work has produced a clearer understanding of how FBXW7 physically interacts with both high-affinity and low-affinity substrates. We review new findings that provide insights into the consequences of “hotspot” missense mutations of *FBXW7* that are found in human cancers. Finally, we discuss how the FBXW7–substrate interaction, and the kinases responsible for substrate phosphorylation, contribute to patterned protein degradation in *C. elegans* development.

## 1. Introduction

Much of the protein degradation performed by eukaryotic cells is accomplished by the ubiquitin–proteasome system (UPS), a mechanism that is highly specific and well-suited to rapid events, such as cell cycle progression, response to extracellular signals, and development. The UPS makes use of post-translational modification, specifically addition of the small protein ubiquitin to lysine residues, to mark proteins intended for degradation [1]. After the initial ubiquitin modification, subsequent additions at lysine residues within ubiquitin itself produces poly-ubiquitin chains. Poly-ubiquitin linked through the ubiquitin residue K48 serves as a protein degradation signal that targets its attached protein for destruction by the proteasome.

Covalent addition of ubiquitin to a protein substrate requires a hierarchy of enzymes: an E1 ubiquitin activating enzyme, an E2 ubiquitin conjugating enzyme, and an E3 ubiquitin ligating enzyme. The ultimate step in this pathway, the E3 enzyme, is responsible for binding the targeted substrate and directly or indirectly catalyzing its modification with ubiquitin [2]. Eukaryotes have a very small number of genes encoding E1 enzymes (typically 1–2) and E2 enzymes (typically a few dozen). In contrast, there are hundreds of eukaryotic genes encoding E3 enzymes, a complexity that permits individualized recognition, binding, and modification for a broad diversity of cellular proteins. Cullin–RING ligases (CRLs) constitute a superfamily of E3 ubiquitin ligases defined as multi-protein complexes consisting of a Cullin protein, a RING protein, and a substrate recognition subunit [3]. Within CRLs, it is common for the substrate recognition subunit to be comprised of multiple proteins. For example, CRLs containing CUL1 act with a substrate recognition complex containing SKP1 and an F-box protein, a complex termed Skp1–Cullin1–F-box (SCF) [4].

FBXW7 is a highly conserved F-box protein whose function is critical to regulate cell cycle progression, cell signaling, and development. Its requirement for these processes is evident from the discovery of FBXW7 mutants in multiple model organisms, and consequently, this highly conserved protein is referred to by multiple names. These include Cdc4 in budding yeast, Ago in *Drosophila melanogaster*, and SEL-10 in *Caenorhabditis elegans*. In human cells, FBXW7 has tumor suppressor functions and the gene is frequently mutated or silenced in cancers [5]. Since its discovery, the molecular mechanism of substrate recognition by FBXW7 has been the subject of intense investigation. This review discusses how FBXW7 substrate recognition impacts disease and development; specifically, the effects of high- and low-affinity substrate binding, the consequences of missense mutations that alter substrate recognition, and the possible effects of high- and low-affinity substrate interactions during development.

## 2. FBXW7 Binds A Cdc4 Phospho-Degron (CPD)

**Figure 1:**
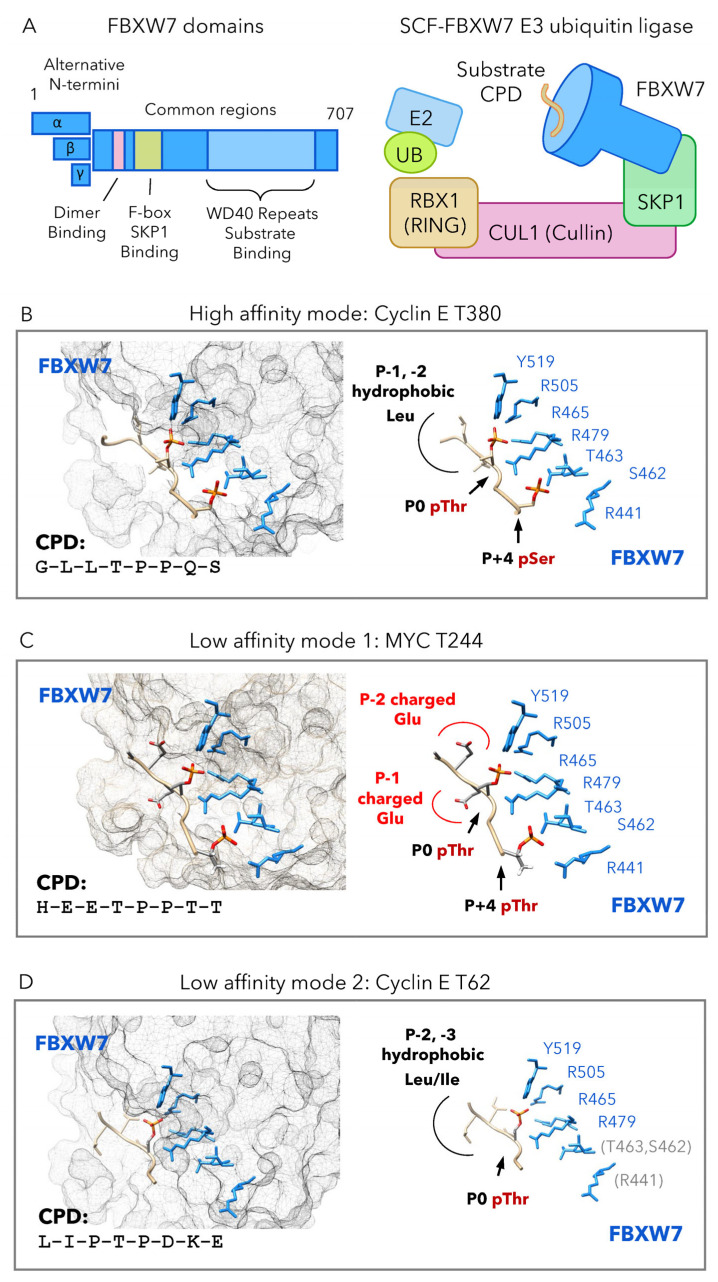
FBXW7 interaction with high and low-affinity substrates. (**A**) Domains of the FBXW7 protein include three different N-termini produced from alternative isoforms and common regions that include the dimerization domain, F-box domain, and WD40 repeat domain. The SCF-FBXW7 E3 ubiquitin ligase is a complex comprising FBXW7, SKP1, CUL1, and RBX1. This complex is capable of recruiting an E2 ubiquitin ligase through RBX1 and CUL1. FBXW7 acts as the substrate recognition component, binding a substrate CPD. (**B**–**D**) Examples of FBXW7 binding with high and low affinity. At left, the FBXW7 WD40 repeat domain surface is semi-transparent, with sidechains involved in CPD binding shown (blue). The CPD peptide (tan) sidechains are shown for P-2, P-1, P0, and P+4 positions. Red is used to indicate negative charges. At right, FBXW7 binding residues (blue) and CPD (tan) are shown without the surface rendering. (**B**) The high-affinity CPD at Cyclin E T380 contains three components: P-2, P-1 hydrophobic leucines, P0 pThr, and P+4 pSer. Binding is coordinated by FBXW7 residues Y519, R505, R479, R465, T463, S462, and R441. (**C**) The low-affinity CPD at MYC T244 has negatively charged glutamates rather than hydrophobic residues at P-2 and P-1. (**D**) The low affinity CPD at Cyclin T62 does not contain a phosphorylated P+4 residue. In this structure, positions after P+1 were disordered. Illustrations were generated from PDB accessions 2OVQ, 7TY1, 2OVR [10,14].

From yeast to humans, FBXW7 proteins share three conserved domains: a dimerization domain required for interaction of two FBXW7 protomers, an F-box domain that mediates FBXW7 interaction with SKP1, and a substrate-binding domain composed of eight WD40 repeats (Figure 1A). The human *FBXW7* gene permits expression of three isoforms that differ in their N-terminus: FBXW7α is localized to the nucleus; FBXW7β to the cytoplasm; FBXW7γ to the nucleolus [6,7]. However, all human isoforms share common regions containing the conserved dimerization domain, F-box, and WD40 repeats. The quaternary structure of an SCF-FBXW7 complex includes the proteins FBXW7, SKP1, CUL1, and RBX1 [8] (Figure 1A). This complex recruits an E2 conjugating enzyme and ubiquitin through the RBX1 and CUL1 proteins. A protein substrate intended for ubiquitination is recruited through the FBXW7 WD40 repeat domains.

FBXW7 exhibits a precise ability to bind and promote degradation of specific proteins. Extensive analyses of a yeast substrate, the cell cycle inhibitor Sic1, and a human substrate, Cyclin E, revealed that the FBXW7 WD40 domain interacts with highest affinity to a doubly phosphorylated substrate peptide, a sequence now termed the Cdc4 phospho-degron (CPD) [9,10]. Since that definition, a large number of high-affinity CPDs in other substrates have been discovered [5,11]. At its simplest, the consensus sequence is pThr-Pro-Pro-X-pSer, where the threonine and serine residues are phosphorylated and X indicates that any amino acid is tolerated. The pThr, sometimes termed the “central threonine”, is located at position P0. Other positions amino-terminal to this are referred to as P-3, P-2, and P-1, while positions at the carboxy terminal are referred to as P+1, P+2, P+3, and P+4.

Phosphorylation of the P0 Thr within a CPD peptide is essential for its interaction with FBXW7 [9], while phosphorylation of the P+4 Ser greatly enhances binding affinity (as reviewed in the following section). An important consequence of this is that substrate binding, ubiquitination, and degradation is necessarily driven by the activity of protein kinase(s) responsible for phosphorylating them. In many cases, the P0 and P+4 sites are phosphorylated by at least two different kinases. This is case for a CPD centered at T380 within human Cyclin E, perhaps the most well-characterized FBXW7 substrate. This motif is first phosphorylated by CDK2 at the P+4 position, then subsequently by GSK3 at the P0 position [12]. GSK3 is well suited for this role as the second kinase because its substrate recognition requires a pre-existing “priming” phosphorylation at a P+4 position [13]. While the kinases needed for P+4 phosphorylation can differ between substrates, GSK3 appears to be responsible for P0 phosphorylation in most FBXW7 substrates [11].

## 3. High Affinity Degron Binding

The eight WD40 repeats of FBXW7 form a beta-propeller domain with binding pockets that accommodate the specific residues and phosphates found in a CPD peptide [8,10]. Sequence similarities between strong high-affinity CPDs establish three sequence characteristics needed for high affinity binding: (i) hydrophobic residues, typically leucine or isoleucine at P-1 and P-2, (ii) pThr-Pro sequence at P0 and P1 positions, and (iii) pSer at P+4. 

A high affinity mode of FBXW7-CPD binding is exemplified by a CPD found at T380 of human Cyclin E (Figure 1B). This CPD is required for normal degradation of Cyclin E protein, making a substantial contribution to its turnover [15]. When complexed with a phospho-peptide containing the Cyclin E T380 CPD, residues of the FBXW7 WD40 repeats make charge-stabilized hydrogen bonds with the CPD pThr and pSer groups. The CPD pThr at P0 position is coordinately bound by four conserved FBXW7 residues located in a shallow, positively charged pocket near the center of the WD40 beta-propeller. In a similar manner, the CPD pSer at P+4 position is bound by four FBXW7 residues, although these interactions occur on a solvent-accessible surface rather than within a pocket [10]. Two other regions of the CPD make contact with FBXW7: leucine residues at P-1 and P-2 positions, and proline residues at P+1 and P+2 positions. It is possible that the prolines at P+1 and P+2 are important for their contacts with FBXW7 as well as the correct spacing and orientation of the pThr and pSer [10]. 

## 4. Low Affinity Degron Binding

In contrast to high-affinity CPDs, many degrons first characterized in the yeast substrate Sic1 are far weaker in binding FBXW7 [9,10]. Many experimental efforts have been directed towards prediction or identification of high-affinity, consensus degrons. This focus is understandable because a single, high-affinity consensus CPD sequence is sufficient to confer FBXW7 binding. However, low-affinity CPDs are found in bona fide FBXW7 substrates, including in human Cyclin E and MYC proteins, which each contain at least two CPDs: one that strongly binds FBXW7, and one that is much weaker. Although these CPDs do not match the established consensus, they functionally contribute to Cyclin E and MYC ubiquitination and degradation, making it important to understand how FBXW7 binds these low-affinity sites.

Structural studies of FBXW7 interaction with human Cyclin E and MYC suggest there are at least two different modes in which FBXW7 binds low-affinity CPDs. The first, referred to here as low-affinity mode 1, was recently discovered in a degron located at T244 of human MYC [14] (Figure 1C). This CPD differs from the consensus in that it has two charged glutamates at P-2 and P-1, positions where the consensus favors hydrophobic residues. Additionally, this CPD contains pThr rather than pSer at the P+4 position. Even when doubly phosphorylated at both P0 and P+4 positions, the MYC T244 CPD binds to FBXW7 with far lower affinity (>20-fold) compared to a consensus CPD located at MYC T58. Binding of the two pThr groups is coordinated within the same charged binding pockets, involving the same FBXW7 residues, used for a high-affinity CPD. The reduced affinity for FBXW7 binding this CPD appears to be a result of interactions at P-2 and P-1 positions, where the MYC T244 CPD has negatively charges [14].

A second low-affinity binding mode, referred to here as binding mode 2, was the first explored in structural studies. This mode is exemplified by a degron located at T62 of human Cyclin E [10] (Figure 1D). The Cyclin E T62 CPD differs from the consensus sequence in that it cannot be phosphorylated at the P+4 position. Additionally, this CPD lacks a P+2 proline, a residue thought to stabilize orientation and contacts within high-affinity CPDs. When complexed with a phospho-peptide containing the Cyclin E T62 CPD, residues of the FBXW7 WD40 beta-propeller coordinate binding of the CPD P0 pThr in the same positively charged pocket used for high-affinity CPDs. However, loss of the P+4 pSer in this degron results in loss of all FBXW7-CPD interactions beyond the P+1 position, accounting for the much lower binding affinity [10].

The FBXW7 dimerization domain is found in orthologs from yeast to human and is required for dimer formation [16,17]. Generally, dimerization increases the ability of FBXW7 to bind and promote ubiquitination of substrates containing low-affinity CPDs. For example, a dimer FBXW7 complex binds degrons lacking the consensus hydrophobic residues at P-2 and P-1 positions, while monomer forms are incapable of doing so [18]. Dimerization allows tolerance of other changes in the CPD; for example, the absence of a P+4 pSer, or the replacement of the P0 pThr with pSer [14,17]. As some substrates are known to have two or multiple CPDs, this raises the intriguing possibility that the FBXW7-substrate interaction is strengthened when the dimer engages with two CPDs in the same substrate.

## 5. *FBXW7* “Hotspot” Mutations

In human cancers, the *FBXW7* gene is frequently silenced or mutated. Mutations are typically loss-of-function in nature, including nonsense changes, truncations, and deletions. Several missense changes in *FBXW7*, termed “hotspot” mutations, recur in a number of different cancers, most prominently in T-cell acute lymphoblastic leukemia (T-ALL) [19], but also in others, such as colorectal cancers [20] and melanomas [21]. In T-ALL, *FBXW7* missense mutations primarily alter three amino acids, R465, R505, and R479, which are responsible for coordinating interaction with the substrate CPD pThr at P0. The most common of these three mutations, R465C, disrupts FBXW7 interaction with human NOTCH1 intracellular domain, the relevant substrate in T-ALL [19]. This finding strongly supports the importance of the direct interactions observed in FBXW7-CPD structural models. Additionally, it might suggest that mutation of a charged residue like R465, or others that contact the CPD, would abolish contact with all FBXW7 substrates.

More than 15 years after the discovery of *FBXW7* missense mutations in T-ALL, new findings continue to expand our understanding of their consequences. In all cancers, the hotspot residues R465, R505, and R479 remain the most frequently mutated, as reviewed in [22]. However, careful assessments of these and other recurrent missense mutations have revealed that not all changes are equivalent to R465C in their effects on FBXW7-CPD binding and substrate degradation. Here we review the consequences of *FBXW7* missense mutations on two different substrates, human MYC and BRAF.

## 6. Selective Disruption of a Low-Affinity Binding Mode

T-cell acute lymphoblastic leukemia (T-ALL) is a malignant neoplasm associated with mutations in genes *NOTCH1* and *FBXW7* [23]. In addition to mutations altering FBXW7 residues R465, R505, and R479, the missense mutation R689W is found less frequently in T-ALL [24]. In contrast to FBXW7 residues that coordinate binding with the CPD pThr or pSer, R689 is responsible for stabilizing interactions of the FBXW7 binding pocket with charged groups found only in low-affinity CPDs, specifically glutamate residues at P-2 and P-1 in the low-affinity MYC CPD at T244 [14]. The FBXW7 R689W mutant is rendered incapable of binding the MYC T244 CPD yet retains the ability to bind a high-affinity MYC CPD located at T58. This suggests the possibility that some *FBXW7* mutations selectively disrupt degradation for a subset of protein substrates. It has not yet been tested whether the R689W mutant alters FBXW7′s ability to regulate NOTCH1, a substrate that drives tumorigenesis in T-ALL, but it is notable that the CPD characterized in NOTCH1 also resembles a low-affinity degron [19].

## 7. Unexpected Differences between *FBXW7* Hotspot Mutations

Adult T-cell leukemia (ATL) is a malignancy of T cells caused by human T-cell leukemia virus type 1 (HTLV-I). Like T-ALL, ATL is highly associated with mutations in *NOTCH1* and *FBXW7* [25]. Surprisingly, *FBXW7* missense mutations discovered in ATL affect different residues than those found in T-ALL. Many ATL-associated changes are located within the FBXW7 WD40 repeats, strongly suggesting that they disrupt substrate recognition. For example, one ATL mutation alters FBXW7 residue S462, which directly interacts with the phosphorylated P+4 position of a CPD [10,25] (Figure 1B,C). Among other FBXW7 sites changed in ATL, the residues W425, L443, and H468 are inside or near to the CPD binding pocket. It is not yet understood why T-ALL and ATL, which both affect T cells, are associated with different mutations of *FBXW7*. 

Recent work by Yeh and colleagues investigated the molecular consequences of the ATL-associated *FBXW7* mutation S462P. They discovered that the kinase BRAF is among the most upregulated proteins in cells carrying this mutation, and that its activity promotes ATL tumor resistance to BET inhibitors [26]. They and others found that FBXW7 interacts with BRAF protein to promote its ubiquitination and degradation [26,27]. However, systematic tests of multiple FBXW7 mutants reveal unexpected differences in their ability to bind and regulate BRAF. Some mutations, including those at the T-ALL hotspot residue R465, and the ATL-associated sites S462 and H468, block ubiquitination and degradation of BRAF. Changes to these FBXW7 residues also prevent degradation of other tested substrates, such as NOTCH1, Cyclin E, and MYC. In contrast, mutation of the T-ALL hotspot residue R505 has no effect on BRAF degradation [26]. Along the same lines, work in mouse cells found that mutation of a third hotspot site, R479, also fails to alter BRAF degradation [28]. The BRAF protein contains a CPD centered at T401 [27,29] and has the critical P0 Thr-Pro sequence and P+4 Ser. However, it lacks the leucine or isoleucine residues typically found at P-2 and P-1 positions of high-affinity CPDs. Whether this site is the sole CPD in BRAF, or how its sequence properties permit regulation by some FBXW7 mutants but not others, is still unknown.

## 8. FBXW7 Substrate Degradation in Development

Studies in model organisms, conducted in *C. elegans*, fruit fly, frog, and mouse, demonstrate that FBXW7 has multiple roles in development. For example, FBXW7 was first described in animals through the discovery of a *C. elegans* mutant that altered Notch activity, and consequently, cell fate specification [30]. In *Drosophila melanogaster*, FBXW7 regulates the Cyclin E and MYC proteins in the proliferating cells of developing imaginal discs [31,32]. Many studies show that FBXW7 impacts patterning and cell fate specification; for example, in neural crest and melanocyte development in *Xenopus laevis* [33], trachea branching in *Drosophila melanogaster* [34], and cell fates in hematopoiesis, Sertoli cells, and airway cells of the mouse [35,36,37].

In development, patterned kinase activation presumably drives patterned degradation of FBXW7 substrates. In this context, it is interesting to consider the predicted effects of changing kinase activity. With increasing kinase activity, degradation of a substrate containing a single, high-affinity CPD is expected to respond in a simple manner in which small increases to kinase activity cause substantial increases to substrate degradation [38]. On the other hand, degradation of a substrate with multiple low-affinity CPDs is expected to respond in switch-like manner. In this mode, small increases to kinase activity have little effect until the activity reaches a threshold at which degradation is triggered.

In the following section, we turn to the nematode *C. elegans* to describe two examples of FBXW7 substrates. This animal is optically transparent, making it an advantageous model to monitor FBXW7-regulated protein degradation during live development. Additionally, *C. elegans* mutants lacking the FBXW7 ortholog SEL-10 are viable [39], a contrast to the lethality seen in vertebrates, insects, and even yeast. This has permitted discovery of SEL-10 substrates in many cellular contexts, from the early embryo [40], to neuronal synapses in larval development [41], and oogenesis in adults [42]. 

## 9. *C. elegans* Development: Comparison of High- and Low-Affinity CPDs

**Figure 2:**
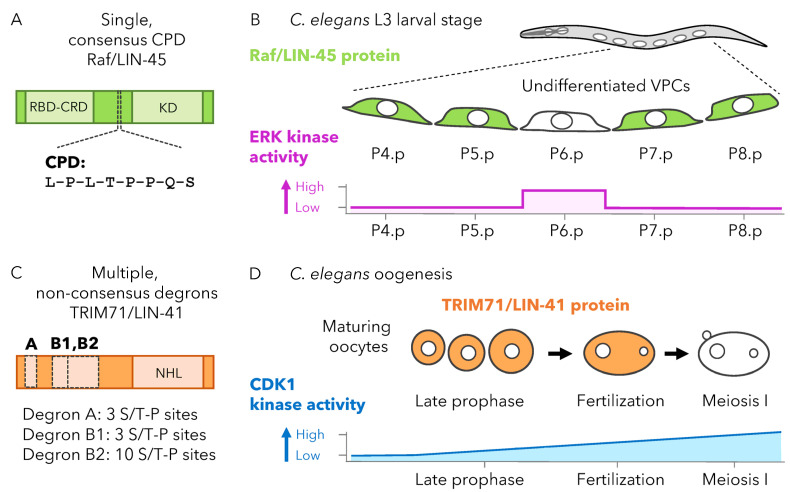
Patterns of FBXW7 degradation for high- and low-affinity substrates. (**A**) The *C. elegans* Raf ortholog LIN-45 contains one known CPD that conforms to t he high-affinity consensus. LIN-45 protein and conserved domains (green), including the Ras-binding domain (RBD), the cysteine-rich domain (CRD), and the kinase domain (KD). CPD sequence is shown below. (**B**) At the *C. elegans* L3 larval stage, a population of cells termed VPCs express LIN-45 protein (green). Although initially expressed in all VPCs, LIN-45 is degraded in one denoted P6.p. Degradation of LIN-45 requires the *C. elegans* FBXW7 ortholog SEL-10 and the kinases ERK, CDK2, and GSK3. At the L3 stage, elimination of LIN-45 closely matches the pattern of ERK activation, with high ERK activation and LIN-45 degradation both occurring specifically in P6.p ERK activation is summarized as low or high for each cell (magenta). (**C**) The *C. elegans* RNA-binding TRIM71 ortholog LIN-41 contains multiple, non-consensus degrons. LIN-41 protein and conserved domains (orange), including the NHL repeats (NHL). Degron A, B1, and B2 are non-overlapping regions required for LIN-41 degradation and enriched in Ser/Thr-Pro sequences. (**D**) During *C. elegans* oogenesis, maturing oocytes in late prophase express LIN-41 protein (orange). LIN-41 is seen in oocytes just after fertilization but is rapidly eliminated as they complete the first division of meiosis. LIN-41 degradation requires SEL-10 and the kinase CDK1. Activation of CDK1 is presumed to increase as oocytes progress from late prophase to the meiosis I division, as summarized for late prophase, fertilization, and meiosis I (blue). In contrast, LIN-41 degradation occurs precisely at the division of meiosis I. Illustrations summarize data from [43,47].

*C. elegans* has a single Raf family ortholog, LIN-45, that is highly similar to BRAF. The LIN-45 protein contains one degron nearly identical to the consensus high-affinity CPD [29]. This CPD, centered at T432 within LIN-45, contains leucines in the P-3 and -2 positions, a P0 Thr-Pro sequence, and a P+4 Ser (Figure 2A). During larval development, LIN-45 protein is initially expressed uniformly in an undifferentiated epithelial cell population termed vulval precursor cells (VPCs). At the L3 larval stage, LIN-45 protein is eliminated in one VPC termed P6.p (Figure 2B), an event that requires SEL-10, as well as the CPD P0 Thr and P+4 Ser residues. LIN-45 degradation requires the kinases ERK, CDK2, and GSK3, and the pattern can be altered by increasing the activation level of either ERK or CDK2 [43]. 

Because LIN-45 contains a single CPD that conforms to the high-affinity consensus, its degradation is expected to respond in a simple manner to kinase activity. This prediction is consistent with the observation that LIN-45 degradation closely matches the spatial activation pattern of ERK, which is specific to P6.p [44], and the temporal activation pattern of CDKs, which are inactive early and activated at the beginning of the L3 larval stage [45,46]. In this model, degradation of a FBXW7 substrate will correspond closely to kinase activation, and patterns of protein degradation will be primarily determined by restriction of kinase activity.

The *C. elegans* NHL/TRIM71 ortholog is termed LIN-41 and contains multiple degrons that resemble known low-affinity CPDs like those found in yeast Sic1 [47] (Figure 2C). LIN-41 protein is abundantly expressed in maturing oocytes of the *C. elegans* hermaphrodite germ line but eliminated in a precisely timed manner during the oocyte-to-embryo transition (Figure 2D). This degradation requires SEL-10 and at least three non-overlapping Ser/Thr-Pro-rich regions within the LIN-41 protein. Mutation analysis of these degrons revealed one Thr-Pro site at LIN-41 T83 required for degradation. However, in striking similarity to yeast Sic1, neither T83 nor any other Ser/Thr-Pro sites contain a P+4 serine or the other critical elements found in high-affinity CPDs. The timing of LIN-41 degradation at the oocyte-to-embryo transition is determined by CDK1 activity [47], which increases as oocytes mature and progress through meiosis. 

Because LIN-41 contains multiple degrons, each with multiple sites that resemble low-affinity CPDs, its degradation is expected to respond to increasing kinase activity in a switch-like manner. This possibility is consistent with the extremely rapid pace of LIN-41 protein elimination, which is completed within a ~10 to 15-min period during the first meiotic division. In this model, degradation of a FBXW7 substrate similar to LIN-41 may occur with sharply defined boundaries or thresholds, even when the pattern of kinase activation is more graded.

## 10. Concluding Remarks

In its role as the recognition subunit of an SCF-FBXW7 E3 ubiquitin ligase, FBXW7 achieves remarkable specificity in substrate binding. It is intriguing that new modes of FBXW7 binding to low-affinity CPDs, such as that described at MYC T244, are now being described. This new information certainly supports the existing definition of high-affinity CPDs. Perhaps more importantly, it expands our view when searching for low-affinity CPDs and raises the possibility that other, still unknown binding modes exist. If we consider the MYC T244 example, it appears possible that substrates governed by low-affinity CPDs may be more strongly impacted by cancer-associated *FBXW7* missense mutations, such as those that alter residue R689. However, there is not yet a simple mechanism to explain how the common hotspot mutations at FBXW7 residues R465, R505, and R479 affect different substrates like BRAF and NOTCH1, unequally. Perhaps this can be better understood with more information on the number and affinity of CPDs in BRAF and NOTCH1. Finally, discoveries of low-affinity CPDs that promote degradation of human Cyclin E, human MYC, and *C. elegans* LIN-41, highlight their contribution to normal substrate regulation, and possibly timing and spatial patterning in development, making it clear that lower affinity does not predict lower importance.

## Data Availability

Not applicable.

## Abbreviations

Ubiquitin–proteasome system (UPS), Cullin–RING ligase (CRL), Skp1–Cullin1–F-box (SCF), Cdc4 phospho-degron (CPD), phospho-threonine (pThr), phospho-serine (pSer), T cell acute lymphoblastic leukemia (T-ALL), adult T cell leukemia (ATL), extracellular signal-regulated kinase (ERK), cyclin-dependent kinase 2 (CDK2), cyclin-dependent kinase 1 (CDK1), glycogen synthase kinase 3 (GSK3).
